# *Vibrio cholerae*-specific antibodies in plasma and saliva in cholera patients during a severe outbreak in Zambia: an antibody profiling approach

**DOI:** 10.3389/fimmu.2025.1641319

**Published:** 2025-08-15

**Authors:** Charlie C. Luchen, Harriet Ngo’mbe, Fraser Liswaniso, Samuel Bosomprah, Julia A. Zhiteneva, Jason Harris, Richelle C. Charles, Edward T. Ryan, David Sack, Biana Bernshtein, Caroline C. Chisenga

**Affiliations:** ^1^ Department of Global Health, Amsterdam UMC, University of Amsterdam, Amsterdam Institute for Global Health and Development, Amsterdam, Netherlands; ^2^ Research Division, Centre for Infectious Disease Research in Zambia, Lusaka, Zambia; ^3^ Department of Biostatistics, School of Public Health, University of Ghana, Accra, Ghana; ^4^ Ragon Institute of Mass General, MIT, and Harvard, Cambridge, MA, United States; ^5^ Division of Infectious Diseases, Massachusetts General Hospital, Boston, MA, United States; ^6^ Department of Pediatrics, Harvard Medical School, Boston, MA, United States; ^7^ Department of Immunology and Infectious Diseases, Harvard T.H. Chan School of Public Health, Boston, MA, United States; ^8^ Department of Medicine, Harvard Medical School, Boston, MA, United States; ^9^ Center for Immunization Research, Johns Hopkins Bloomberg School of Public Health, Baltimore, MD, United States

**Keywords:** cholera, saliva, plasma, antibody, immunology

## Abstract

**Background:**

Cholera is a public health threat in resource-limited settings and is responsible for causing over 3 million cases globally. Mucosal immune responses play an important role in protecting against *Vibrio cholerae* infection, a non-invasive mucosal pathogen, yet traditional plasma-based assays are invasive and logistically challenging, particularly during outbreaks in low- and middle-income countries (LMICs). Saliva offers a unique window into mucosal immunity and may serve as a non-invasive alternative for seroprevalence and vaccine immunogenicity studies.

**Methods:**

We conducted a cross-sectional antibody profiling study to analyse cholera-specific antibodies in saliva and plasma samples from 74 participants upon presenting to the cholera treatment centres. These were collected from four treatment centres in Lusaka during Zambia’s most severe cholera outbreak in 2024 caused by *Vibrio cholerae* O1 Ogawa. Levels of total IgG, IgG1-3, IgM, secretory IgA, and IgA1–2 isotypes were used to compare the biomarker profile between the two sample types.

**Results:**

Saliva and plasma antibody profiles were comparable, with elevated IgA1 and IgA2 responses to cholera toxin-B (CtxB), sialidase, HlyA, and TcpA in saliva. Broader systemic responses were seen in plasma, including high CtxB-specific IgM, IgA1, and total IgG levels. Notably, biomarkers such as HlyA, Ogawa O-specific polysaccharide (OSP), and sialidase exhibited significant positive correlations between plasma and saliva. Elevated biomarker levels of HlyA, Ogawa O-specific polysaccharide (OSP), and sialidase in people living with HIV/AIDS (PLWHA) suggested immunological differences that warrant further exploration.

**Conclusion:**

We demonstrate that saliva is a viable, non-invasive alternative for cholera antibody-based profiling, offering practical advantages in resource-constrained settings. Given its strong correlation with systemic antibody profiles, saliva may be a practical sample for sero-surveillance in resource-limited settings. Future studies should investigate the duration of these salivary responses to further substantiate their use in estimating disease burden and immunity.

## Introduction

Cholera continues to be a major public health challenge, particularly in low- and middle-income countries (LMICs), where recurrent outbreaks place significant strain on health systems. Zambia has reported cholera outbreaks since the early 1970s, with the recent 2024 outbreak being the most severe to date, affecting over 20,000 individuals (1, 2). The predominant serotype during this outbreak was serogroup O1 Ogawa (3). Despite the availability of vaccines, there is no established correlate of protection against *Vibrio cholerae*, thus limiting the ability to predict vaccine efficacy and immunogenicity. Vibriocidal antibody titres correlate with protection and often serve as surrogate endpoints in cholera vaccine trials (4, 5). However, the vibriocidal assay traditionally relies on serum samples, which are invasive and logistically challenging to collect, especially in outbreak conditions, and are labour-intensive in the laboratory.

Alternative non-invasive sample types, such as saliva, could mitigate these barriers (6). Saliva collection is easier and more acceptable in resource-limited settings but has been underexplored in cholera immunology. Our previous study demonstrated the feasibility of using saliva for vibriocidal antibody titres, showing comparable sensitivity and specificity to serum (7). This raises the potential for saliva to be a viable alternative for serological assessments in cholera, as was only shown in an earlier cholera vaccine study (8). However, a comprehensive analysis comparing the broader immunological biomarker profiles in plasma and saliva using advanced multiplexed and sample-sparing antibody profiling approaches remains lacking.

There are limited data on the direct assessment of mucosal anti-*Vibrio cholerae* antibody responses, which leaves a gap in our understanding of protective immune mechanisms at the site of pathogen entry. Although a few studies in vaccinees have analysed stool antibodies and measured day 7 antibody-secreting cell responses, believed to reflect mucosal immunity as maturing lymphoblasts transiently circulate before rehoming to the intestinal mucosa (9, 10), no data are available on salivary mucosal responses in humans with cholera.

Here, we applied an antibody profiling approach on samples collected from participants with cholera to enable high-dimensional profiling of antibody features across multiple antigens in parallel, providing a holistic and unbiased view of immune responses (11, 12). We hypothesised that saliva and plasma would yield comparable profiles of cholera-specific antibodies, while unique antibody signatures found in saliva would enable insights into mucosal immunity. By comparing cholera-specific antibodies between saliva and plasma, we aimed to identify saliva’s utility as an alternative sample for seroprevalence studies and its potential in outbreak settings.

## Materials and methods

### Study design and participant recruitment

We collected samples from four sites in Lusaka in 2024: George Urban Health Centre, Matero Level One Hospital, Levy Mwanawasa General Hospital, and Heroes Cholera Treatment Centre during an ongoing outbreak previously confirmed by the Ministry of Health (MOH) to be due to *Vibrio cholerae* O1 infection. Participants were eligible for enrolment if they presented to one of the four facilities, had watery diarrhoea, and had a positive Cholera Rapid Diagnostic Test (RDT) on the Bioline™ Cholera Ag O1/O139 (Abbot) result. Eligible participants were approached and provided with study information. Those who consented to join the study signed consent forms, with guardians signing on behalf of minors.

We collected approximately 20 ml of blood and 5 ml of saliva from each participant after self-reported onset of diarrhoea and within 24 hours of presentation to the treatment centre. Before saliva collection, participants rinsed their mouths thrice with sterile 20 ml bottled water. Following the mouth rinse, they were then asked to pull and spit saliva into a sterile saliva collection tube with a targeted minimum volume of 5 ml. The collected samples were transported to the Centre for Infectious Disease Research in Zambia (CIDRZ) laboratories at 4–8 degrees the same day. The blood was centrifuged to obtain plasma, which was subsequently screened for HIV using the Determine™ HIV-1/2 kit (Abbot). The remaining plasma and saliva samples were aliquoted and stored at -80 degrees. Within a month of storage, the samples were shipped to Ragon Institute of Mass General, MIT, and Harvard, Cambridge, MA, USA laboratories for antibody profiling on dry ice.

### Antibody profiling

Cholera-specific antibody subclass/isotype levels were assessed using a 384-well-based customised multiplexed Luminex assay, as previously described (13). HlyA, TcpA, CtxB, sialidase, and *V. cholerae* Ogawa O-specific antigen (OSP) were used to profile specific antibody responses, using antigens purified as previously described (14). Tetanus toxin and bovine serum albumin (CEFTA, Mabtech Inc) were used as control antigens. Protein antigens were coupled to magnetic Luminex beads (Luminex Corp) by carbodiimide-NHS ester-coupling (Thermo Fisher). *V. cholerae* Ogawa OSP was modified by 4-(4,6-dimethoxy[1,3,5] triazin-2-yl)-4-methyl-morpholinium and conjugated to Luminex Magplex carboxylated beads. Antigen-coupled microspheres were washed and incubated with plasma or saliva samples at an appropriate sample dilution (IgG: 1:2000 for plasma, 1:20 in saliva; IgA and IgM, 1:1000 in plasma, 1:50 in saliva, samples diluted in 0.1% BSA in PBS) for 2 hours at 37°C in 384-well plates (Greiner Bio-One). Unbound antibodies were washed away, and antigen-bound antibodies were detected by using a PE-coupled detection antibody for each subclass and isotype (IgG1, IgG2, IgG3, IgA1, IgA2, secretory IgA(sIgA) and IgM; Southern Biotech. After one hour of incubation, plates were washed, flow cytometry was performed with an IQue (Intellicyt), and analysis was performed on IntelliCyt ForeCyt (v8.1). All washes were performed with 0.05% Tween 0.1% BSA in PBS. PE median fluorescent intensity (MFI) was reported as a readout for antigen-specific antibody measurements.

### Sample size

We utilised a convenience sampling approach due to the rapid response required during the cholera outbreak and the availability of reagents for antibody profiling. An enrolment size of 74 was targeted to ensure adequate representation across different age groups and sex. While not formally powered for inferential comparisons, this sample size was considered sufficient to provide preliminary insights into cholera-specific antibody responses in plasma and saliva.

### Data analysis

Data analysis was performed using R version 4.4.2. Spearman correlation coefficients were computed to assess the relationship between cholera-specific antibody responses in plasma and saliva. Correlation heatmaps were generated using the corrplot (https://github.com/taiyun/corrplot) and ComplexHeatmap (15) packages to visualise biomarker median fluorescent intensities across groups. To compare antibody intensities between participant subgroups, such as HIV status and age categories, we conducted Wilcoxon rank-sum tests for group comparisons with a significance threshold of p < 0.05. False discovery rate (FDR) correction was applied using the Benjamini-Hochberg method to account for multiple comparisons (16).

### Ethics statement

Before enrolment, all participants provided written informed consent. We sought ethical approval from the University of Zambia Biomedical Research Ethics Committee (UNZABREC), reference number 001-02-23, and the National Health Research Authority (NHRA), which authorised the study. This study was approved by the Massachusetts General Hospital’s Institutional Review Board. All study procedures were conducted under Good Clinical Practice.

## Results

### Participant descriptions

Plasma and saliva samples were obtained from 74 individuals who tested positive for cholera using a Rapid Diagnostic Test (RDT). Of these, 41 were male (55%), with a median age of 19 years; 38 were above the age of 18 (51%), and 9 were persons living with HIV/AIDS (PLWHA, 12%) (**Table 1**). A majority of our participants had missing data on hydration status, limiting meaningful interpretation on the impact of the severity of infection on antibody profiles.

**Table 1:** Patient characteristics.

Variable	Overall	N = 74
Sex	74	
Female		33 (45%)
Male		41 (55%)
Median age in years (IQR)	74	19 (11 – 32)
Age range in years	74	
Under 5		10 (14%)
5 to 18		26 (35%)
Above 18		38 (51%)
HIV status	74	
Negative		65 (88%)
Positive		9 (12%)
Dehydration status	22*^1^*	
Mild Dehydration		20 (91%)
Severe Dehydration		2 (9.1%)

*^1^* not equal to 74 due to missing data on dehydration

IQR, Interquartile range; N, number of participants; data on HIV status was collected by testing using the Determine™ HIV-1/2 kit (Abbot)

### Antibody profiles across plasma and saliva

**Figure 1:**
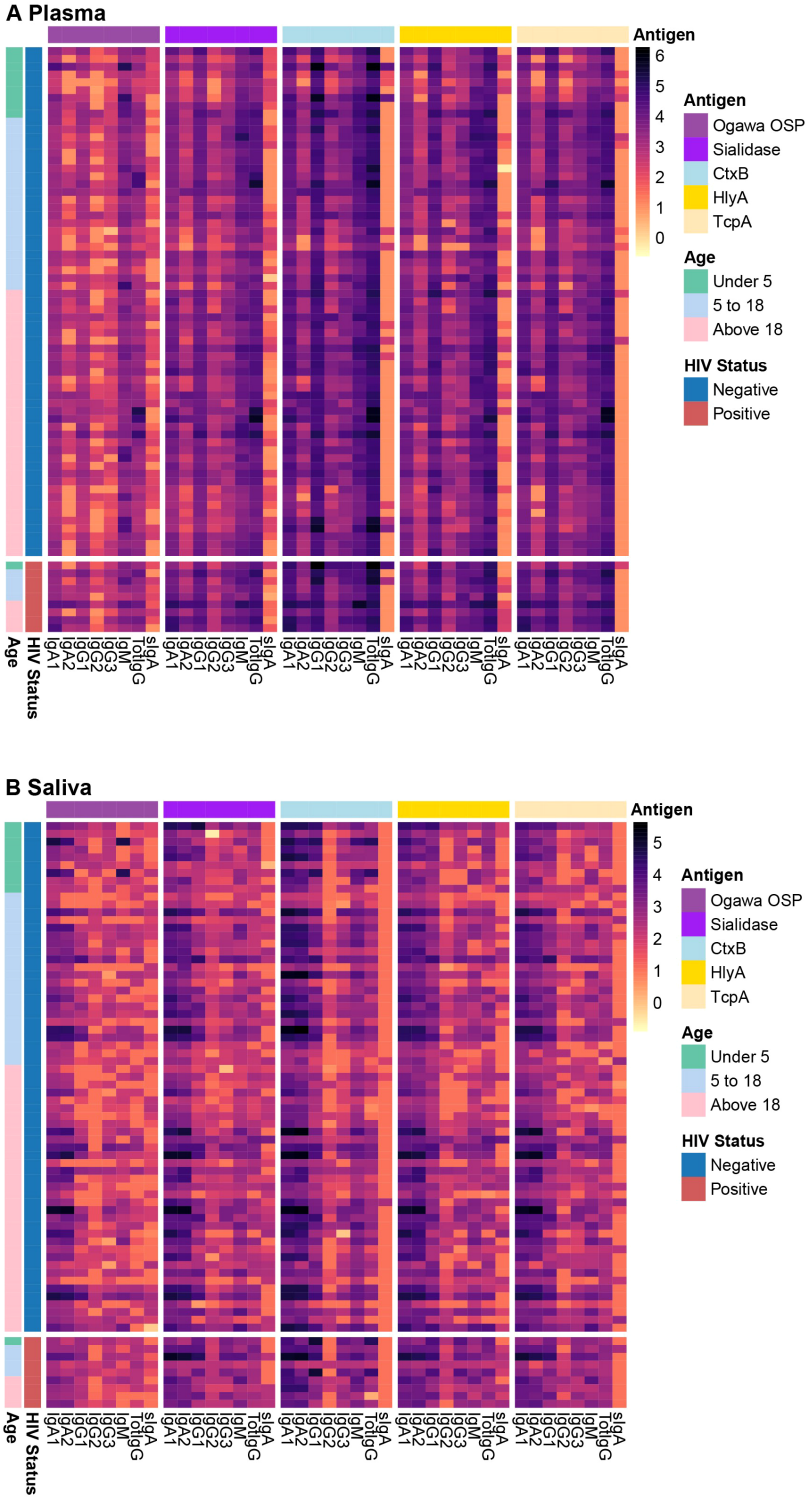
Plasma and saliva antibody profiles. The heatmap shows antibody profiles between **(A)** plasma and **(B)** saliva grouped by HIV status and age ranges in years. The rows represent individual samples, and colours represent the magnitude of antibody responses (log 10), with darker shades corresponding to higher response levels.

We first explored the distribution of raw antibody subclasses specific to key *V. cholerae* antigens, including sialidase, CtxB, HlyA, TcpA, and Ogawa OSP across the different sample types. A brief description of these antigens is in the **Supplementary Table 1**. All participants in this study were infected with *V. cholerae* O1, serotype Ogawa. Antibody responses in plasma were predominantly IgG, while in saliva IgA was the predominant antibody isotype, across different antigens. Moreover, while IgA2 levels in plasma were lower than IgA1, in saliva levels of both IgA isotypes were comparable. Nonetheless, we detected IgA in plasma and IgG in saliva as well (**Figure 1**, **Supplementary File 1**).

### Correlation of antibody responses in plasma and saliva

We were then interested in assessing the interchangeability of biomarkers between plasma and saliva. For this, we performed Spearman correlation analyses for antibody responses across the two sample types. This approach allowed us to identify biomarkers consistently correlated in systemic and mucosal responses.

**Figure 2:**
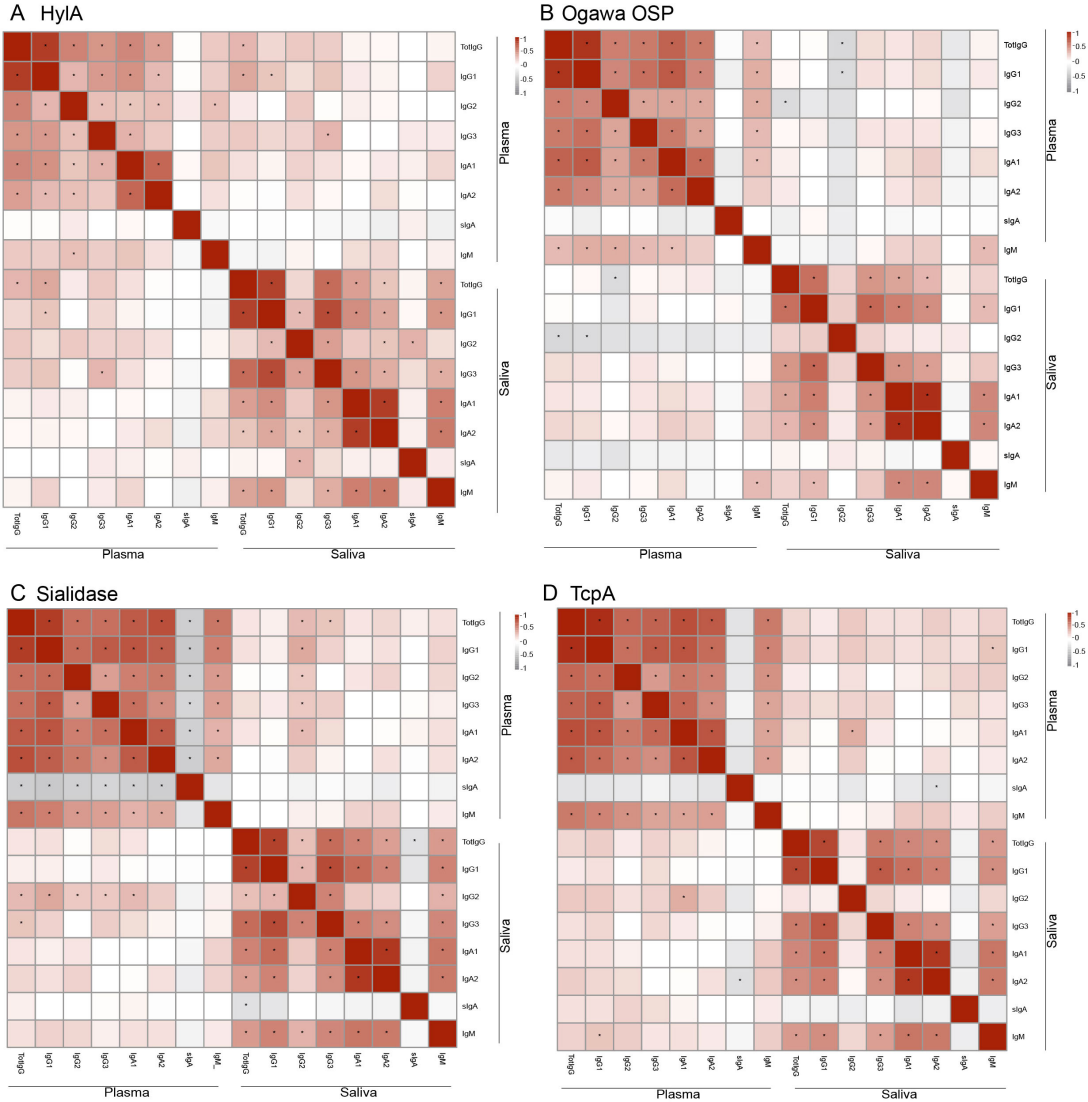
Correlation of biomarkers between plasma and saliva. **(A)** correlation of HylA subtypes. **(B)** correlation of Ogawa OSP subtypes. **(C)** correlation of Sialidase subtypes. **(D)** correlation of TcpA subtypes. The colours indicate Spearman correlation values, with positives shown in red and negatives in grey. *Show significant differences (*p* < 0.05).

We observed that most immunological biomarkers exhibited positive correlations between plasma and saliva antibody levels. Notably, significant correlations were found for HlyA-specific responses across total IgG, IgG1, and IgG3 isotypes (**Figure 2A**). Also, the Ogawa OSP IgM response showed strong positive correlations between the two sample types (**Figure 2B**). Additionally, sialidase IgG2 levels showed significant positive correlations in both plasma and saliva, with its levels in saliva positively associated with all IgG subtypes and IgA1 in plasma (**Figure 2C**). In contrast, no significant correlations were observed between plasma and saliva for TcpA and CtxB-specific responses across the same antibody subclasses (**Figure 2D**, **Supplementary Figure 1**). However, we observed a significant positive correlation between IgG2 levels in saliva and IgA1 in plasma for TcpA (**Figure 2D**).

### Association of biomarkers with HIV status

Since 9 of our 72 participants tested positive to HIV, we explored the relationship between HIV status and the biomarkers identified as significantly correlated between plasma and saliva across the entire cohort. All 9 participants living with HIV were adults.

**Figure 3:**
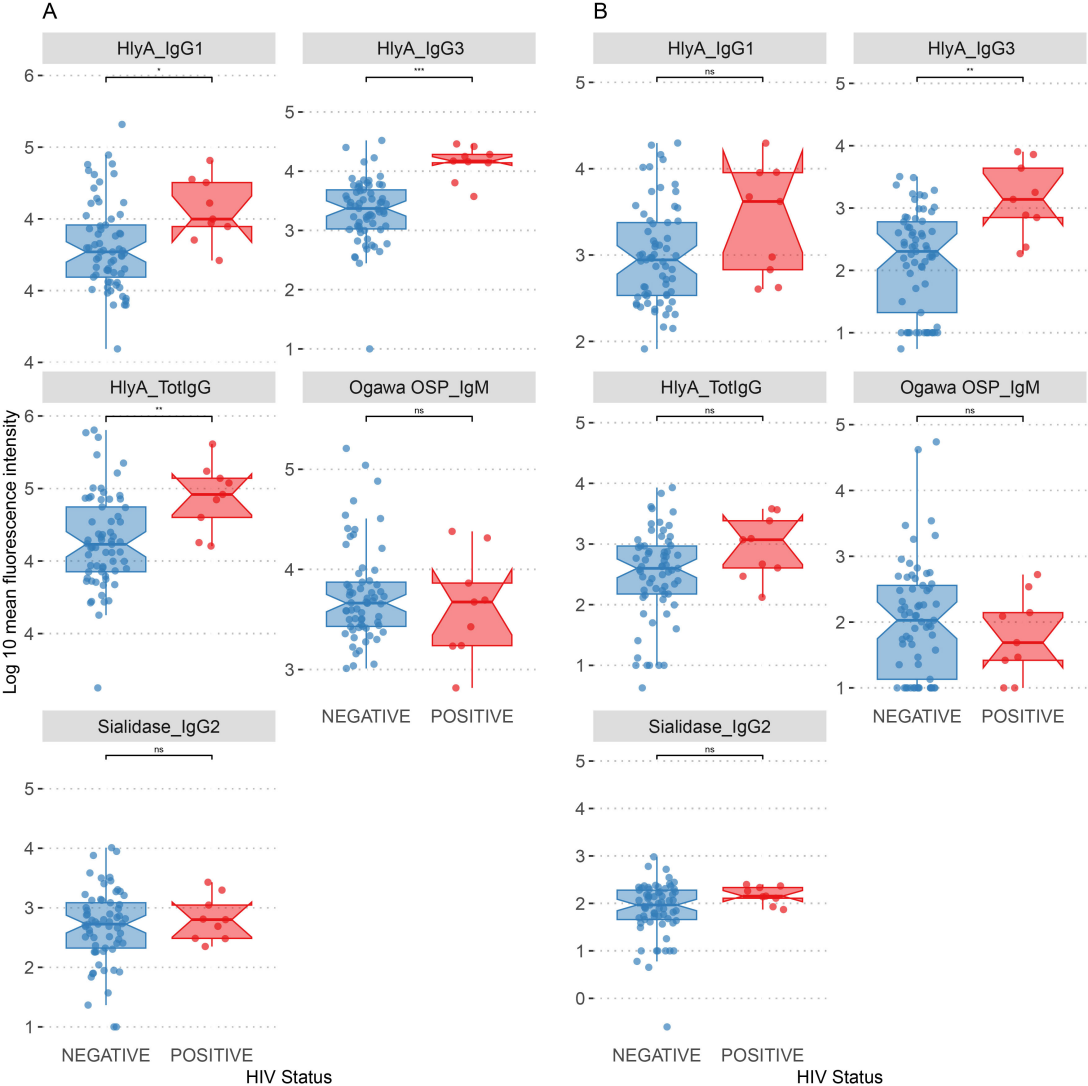
Distribution of biomarkers by HIV status. **(A)** Association of plasma biomarkers by HIV status. **(B)** Association of saliva biomarkers by HIV status. *p.adj < 0.05, **p.adj < 0.01, ***p.adj < 0.001, ns, not significant.

In plasma samples, people living with HIV/AIDS (PLWHA) showed significantly higher levels of HlyA-specific antibodies across multiple antibody classes, including IgG1 (log10 median: HIV-negative = 4.27, HIV-positive = 4.50, *p.adj* < 0.05), IgG3 (log10 median: HIV-negative = 3.37, HIV-positive = 4.17, *p.adj* < 0.001), and total IgG (log10 median: HIV-negative = 4.62, HIV-positive = 4.96, *p.adj* < 0.01). Conversely, no significant differences were observed for Ogawa OSP (IgM) and sialidase (IgG2), although both biomarkers were elevated in PLWHA (**Figure 3A**).

Most of the biomarkers in saliva did not differ significantly between the two groups. Notably, HlyA (IgG3) was the only biomarker that showed a significant increase in PLWHA (log10 median: HIV-negative = 2.30, HIV-positive = 3.14, *p.adj* < 0.05), suggesting an association of this antibody isotype in saliva samples with HIV status (**Figure 3B**).

### Association of biomarkers with participant age

To investigate the influence of age on the biomarkers previously identified as significantly correlated in plasma and saliva, we examined age-related differences in antibody profiles. As demonstrated earlier, we excluded samples from PLWH because of their confounding effects. The analysis revealed distinct patterns in biomarker levels associated with participant age.

**Figure 4:**
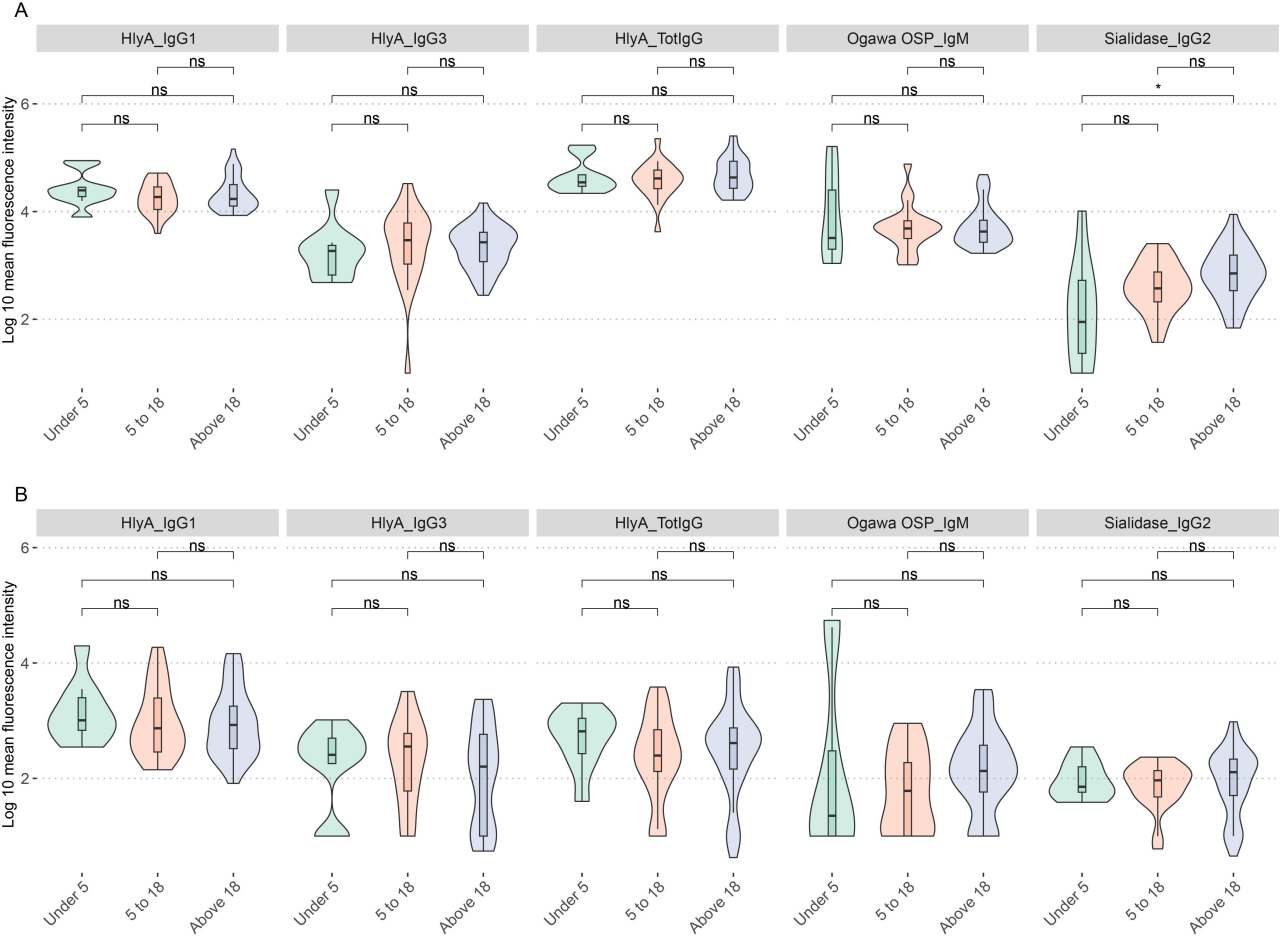
Distribution of biomarkers by age. **(A)** Age differences in biomarker distribution in plasma samples. **(B)** Age differences in biomarker distribution in saliva samples. ns show nonsignificant relationships while *represents significant differences *p.adj* < 0.05.

Trends in age-related differences were observed for most biomarkers in both plasma and saliva samples. Significant differences in plasma sialidase IgG2 levels were observed between participants under 5 years and those above 18 years, with mean fluorescence intensities of 1.98 and 2.695, respectively (*p.adj* < 0.05). Additionally, both age groups exhibited more significant variability in biomarker levels than other age categories (**Figure 4A**). Notably, all age groups showed higher variability in HlyA-specific antibody levels across IgG1 and total IgG. Similar trends were observed in saliva samples (**Figure 4B**) with no age group showing significant differences.

### Antibody profiles previously found to correlate with protection against cholera

**Figure 5:**
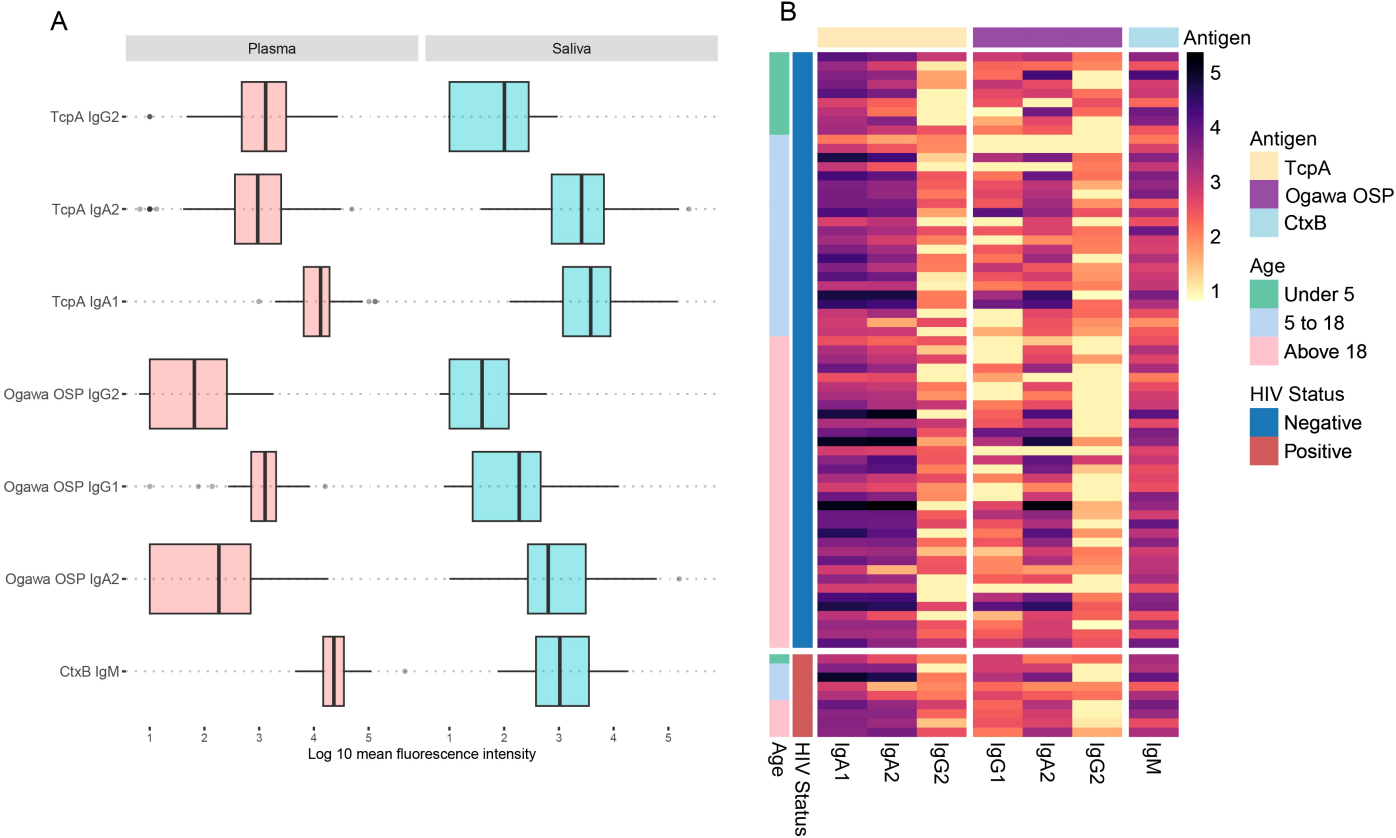
Antibody responses for composite immune responses previously found to correlate with protection against cholera in a Bangladeshi cohort. **(A)** Shows protective antibodies in plasma and saliva. **(B)** Heatmap showing the protective antibodies in saliva, grouped by HIV status and age ranges in years. The rows represent individual samples, and colours represent the magnitude of antibody responses (log 10), with darker shades corresponding to higher response levels.

We then aimed to determine whether antibody profiles associated with protection against *V. cholerae* O1 infection could be detected in both saliva and plasma, as previously reported by Wiens et al. (2023) (14). We detected four key biomarkers in both sample types, namely CtxB IgM, TcpA IgG2, Ogawa OSP IgG1, and TcpA IgA1/2, which Wiens et al. (2023) showed outperform vibriocidal titres and are associated with a lower longitudinal risk of becoming infected (**Figure 5**). However, we did not assess antibody-dependent complement deposition (ADCD), another reported marker correlated with lower risk of infection, as it was not included in our screening protocol. Further analysis of these protective biomarkers in saliva showed high intensities of TcpA IgA1/2 and CtxB IgM across most samples, further highlighting their potential role in mucosal immunity (**Figure 5B**).

## Discussion

In this study, we report that cholera-specific plasma biomarkers can be detected in saliva. To the best of our knowledge, this is the first study to demonstrate a comprehensive multiplex approach of cholera-specific biomarkers in both plasma and saliva. We show differences in how some biomarkers are represented in systemic versus mucosal compartments, such as the dominance of IgG in plasma and IgA in saliva, suggesting that these antigens may elicit compartment-specific immune responses. In addition, identifying robust correlations between specific biomarkers in plasma and saliva, such as HlyA, Ogawa OSP, and sialidase, indicates saliva’s viability as an alternative sample to plasma. Saliva collection is less invasive and more culturally acceptable in field settings, particularly in LMICs like Zambia, where logistical challenges often hinder plasma collection.

Our findings align with earlier studies demonstrating the feasibility of saliva-based antibody testing for cholera (7, 8). However, using an antibody profiling approach to provide a multiplexed and high-dimensional analysis of antibody biomarkers in plasma and saliva represents a novel contribution. While prior studies have primarily focused on plasma biomarkers (4, 14), this research bridges a critical gap by validating saliva as a potential alternative sample type. Moreover, the observed compartment-specific immune responses point to saliva’s unique insights into mucosal immunity, complementing systemic analyses.

We report a positive correlation between HlyA-specific antibodies in both plasma and saliva. HlyA is a toxin secreted by gram-negative bacteria, such as strains of *Vibrio cholerae*, which plays a critical role in pathogenesis and immune responses during infection. It is involved in innate immunity following infection, which can trigger the release of pro-inflammatory cytokines such as IL-1β (17). While less attention has been given to HlyA in cholera immunogenicity studies, a prior study showed that the levels of hemolysin A were correlated with the severity of *Vibrio cholerae* infection in nematodes (18). Current whole-cell-killed vaccines typically target primarily the O-specific polysaccharide of *V. cholerae* O1. Thus, HlyA could serve as a supplementary immunogen to enhance vaccine effectiveness.

We also observed high levels of sialidase antibodies in plasma and saliva, a neuraminidase that facilitates the binding of cholera toxin to epithelial cells. Previous studies report that sialidase plays a role in cholera immune responses and has been associated with protection against cholera infection (19). An intriguing observation was the positive correlation of IgG2 in saliva with IgA1 in plasma. IgG2 subclass plays an important role in immune responses to carbohydrate antigens such as lipopolysaccharides present in *V. cholerae*; thus, its presence in saliva may reflect mucosal immune activation (20). On the other hand, IgA1, the predominant IgA subclass in plasma, has been associated with protection against cholera and is involved in mucosal defence (21). Although we may not fully understand this relation, this may suggest immunological cross-talk between systemic and mucosal compartments, where IgG2 could serve as a surrogate marker for plasma IgA1 responses. We observed a positive correlation in Ogawa-specific IgM in both sample types. Ogawa IgM levels are important in predicting recent *V. cholerae* infection, hence this may be a useful biomarker in outbreak settings for identifying recent infections to track disease transmission.

Immunocompromised individuals present a clinically important subgroup in cholera immunogenicity. One such group is PLWHA, where the epidemiological consequence of the coexistence of HIV and cholera is sparse in endemic regions. Our earlier study showed that individuals who were living with HIV had lower vibriocidal antibody titres compared to individuals not living with HIV following vaccination (22). In this study, we show a trend that PLWHA have higher responses to biomarkers that were positively correlated between plasma and saliva. We report significantly higher plasma levels of HlyA-specific IgG1, IgG3 and total IgG, alongside a rise in salivary HlyA-IgG3 in PLWHA. A plausible explanation could be that HIV infection is frequently accompanied by gingivitis, periodontitis, aphthous ulceration and oral candidiasis, all of which compromise epithelial integrity, potentially increasing both antigen ingress and leakage of plasma-derived antibodies into saliva (23–25). Interestingly, our results suggest that antibodies in saliva could be a more stable readout as they were seemingly not influenced by HIV status. However, since these measurements were taken at a single timepoint, additional longitudinal sampling would be required to determine whether the heightened HlyA response reflects prolonged shedding or has functional protective value and to confirm the stability of the biomarkers in saliva. Beyond HIV status, our age-stratified analysis did not report any significant differences in plasma and saliva samples but for sialidase, which showed significant differences in its presence by age. This observation has been reported previously, in which adults were more likely to have sialidase responses in plasma than children (26, 27).

Finally, identifying biomarkers in saliva, previously reported to be important in cholera infection, is a significant finding. We observed high antibody intensities in saliva for two major *V. cholerae* antigens, TcpA and CtxB. TcpA, an adhesion factor important in intestinal colonisation, has been associated with protective immunity (14). Given its strong intensity in saliva, TcpA-specific IgA1/2 could serve as a useful non-invasive marker of cholera infection. On the other hand, while CtxB is a critical virulence factor in *V. cholerae* pathogenesis, its role in protection remains unclear. While IgM is not the predominant immunoglobulin in saliva, low but measurable amounts are routinely detected. Polymeric IgM may be actively transported across the mucosal epithelium via the polymeric−Ig receptor, while serum−derived IgM can transude through crevicular fluid during acute inflammation (28). Thus, detection of CtxB−specific IgM may reflect an early mucosal response, making it a potential non−invasive marker of acute cholera. Although Haney et al. (2018) (29) did not find significant correlations between CtxB responses and protection in a human challenge model, these responses were associated with protection against cholera infection in a cohort study conducted in Bangladesh (14). This discrepancy may suggest that CtxB’s direct role in protection requires further investigation to confirm its relevance as a correlate of protection. We also observed high levels of Ogawa OSP IgM in saliva, an important marker of protective immunity, which has been reported to cross-react with Inaba OSP (30). Thus, elevated salivary Ogawa OSP IgM, could serve as a plausible non-invasive marker of a recent infection.

While we provide compelling evidence for saliva-based antibody profiling following cholera infection, our study has some limitations. Firstly, the small sample size, though powered for the initial analysis, limits the generalizability of our results. Also, future work should expand biomarker coverage to include other virulence and structural antigens such as OmpV, CtxA, and flagellar proteins, which may elicit mucosal responses relevant to infection and immunity. The variability in biomarker sensitivity across age groups and the incomplete hydration status data further emphasise the need for larger and more comprehensive studies. Additionally, a longitudinal study would have allowed us to track immune responses over time instead of the cross-sectional design employed here. Lastly, while we included PLWHA, a more detailed analysis of immune suppression effects, including stratification by CD4 count and viral load, would provide deeper insights into how mucosal immunity is altered in immunocompromised populations. Future studies should address these limitations to strengthen saliva-based sero-surveillance and vaccine evaluation.

The adoption of saliva-based diagnostics could significantly enhance outbreak preparedness and response strategies by enabling a rapid and non-invasive sample collection in both clinical and community settings. Saliva collection is more suitable for vulnerable populations, including children, and can be scaled rapidly during cholera outbreaks. Additionally, this approach reduces the need for specialised equipment and trained personnel, as required in plasma collection, potentially lowering overall costs and increasing accessibility in remote regions. These advantages align with the global health goals of equitable access to practical diagnostic tools.

## Data Availability

The raw data supporting the conclusions of this article will be made available by the authors, without undue reservation.
