# Supplementary material for: Vibrio cholerae-specific antibodies in plasma and saliva in cholera patients during a severe outbreak in Zambia: an antibody profiling approach
Source: Front Immunol. 2025 Aug 15;16:1641319. doi: 10.3389/fimmu.2025.1641319 (PMC12394519; doi:10.3389/fimmu.2025.1641319)
Supplement: Supplementary file 2 [file Table1.docx]

**Supplementary Table 1.** Functional description of antigens

| Antigen name | Abbreviation | Function |
| --- | --- | --- |
| sialidase | N/A | A neuraminidase that facilitates binding of cholera toxin to intestinal epithelial cells |
| Cholera toxin B-subunit | CtxB | Facilitates entry of the toxin into cells via endocytosis and plays a role in membrane remodelling |
| Haemolysin A | HlyA | A cytolytic protein that exerts haemolytic activity, causing lysis of eukaryotic cells by disrupting the cell membrane |
| Toxin-coregulated pilus A | TcpA | Needed for bacterial aggregation and subsequent colonisation in the host intestine |
| Ogawa O-specific polysaccharide | Ogawa | Confers serogroup, Ogawa, specific immunity |

**Supplementary Figure 1.** Correlation of CtxB subtypes. The colours indicate Spearman correlation values, with positives shown in red and negatives in grey. * Show significant differences (*p* < 0.05).
