# Supplementary figures and images for: Vibrio cholerae-specific antibodies in plasma and saliva in cholera patients during a severe outbreak in Zambia: an antibody profiling approach
Source: Front Immunol. 2025 Aug 15;16:1641319. doi: 10.3389/fimmu.2025.1641319 (PMC12394519; doi:10.3389/fimmu.2025.1641319)

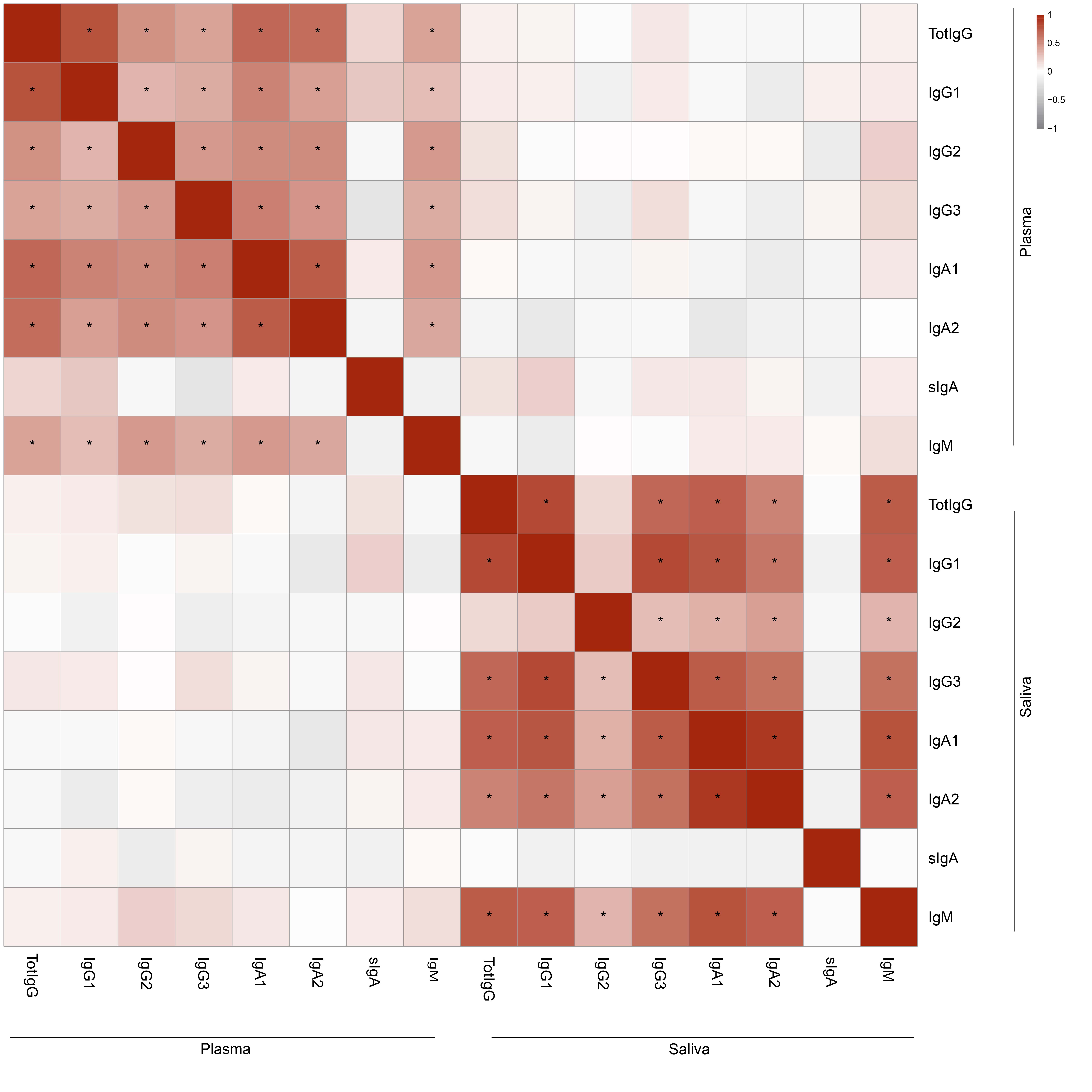

Supplement: Supplementary file 3 [file Image1.jpeg]
